# Research on improved evidence theory based on multi-sensor information fusion

**DOI:** 10.1038/s41598-021-88814-3

**Published:** 2021-04-29

**Authors:** Zhen Lin, Jinye Xie

**Affiliations:** 1 Institute of Technology, Sanya University, Sanya, 572000 China; 2 Sanya University, Sanya, 572000 China

**Keywords:** Computational science, Information technology

## Abstract

In view of the lack of effective information fusion model for heterogeneous multi-sensor, an improved Dempster/Shafer (DS) evidence theory algorithm is designed to fuse heterogeneous multi-sensor information. The algorithm first introduces the compatibility coefficient to characterize the compatibility between the evidence, obtains the weight matrix of each proposition, and then redistributes the basic probability distribution of each focal element to obtain a new evidence source. Then the concept of credibility is introduced, and the average support of evidence credibility and evidence focal element is used to improve the synthesis rule, so as to obtain the fusion result. Compared with other algorithms, the proposed algorithm can solve the problems existing in DS evidence theory when dealing with highly conflicting evidence to a certain extent, and the fusion results are more reasonable and the convergence speed is faster.

## Introduction

In the greenhouse intelligent control, in order to accurately judge the environmental conditions in the greenhouse, it is necessary to use the multi-sensor information fusion technology to integrate the environmental information of the greenhouse, so as to provide the greenhouse managers with accurate environmental information.

At present, there is no effective fusion model and algorithm for information fusion of heterogeneous multi-sensor information^1–3^, but with the help of modern statistical theory, this problem can be effectively solved to a certain extent^4–8^. As an important branch of statistical theory, DS evidence theory is widely used in the field of multi-sensor information fusion due to its ability to represent uncertain and unknown information clearly and its strong operability^9–12^. Although Dempster's rule of combination in this theory can reasonably combine the evidence and fuse the expected experimental results, it has a high requirement on the degree of evidence conflict. When the degree of conflict is high, the fusion results are often inconsistent with the actual situation, and some fusion results even seriously violate common sense^13–15^.

DS evidence theory was first proposed by Dempster and then further developed by his student Shafer, which is an uncertain reasoning method with confidence function as the metric standard. Because DS evidence theory has general conflict, one-vote rejection, robustness and failure of synthesis rules, it will make the results of evidence synthesis deviate from the fact or fail to be synthesized, and then lead to wrong reasoning results, affecting the reliability of fusion results. Therefore, it is necessary to improve DS evidence theory and form an improved DS evidence theory that can overcome the above four problems, so as to form a fusion result that conforms to common sense and can provide decision makers with higher reliability.

The algorithm in this paper can be used to accurately judge the environmental condition of greenhouse and implement the corresponding control measures, so that greenhouse crops can grow in a suitable environment.

## Improve DS evidence theory

In this study, an improved DS evidence theory algorithm is designed, which starts from both the improvement of combination rules and the modification of evidence source. First, the compatibility coefficient is introduced to modify the evidence source, and then the reliability and the average support degree of evidence focal elements are introduced to improve the combination rules.

### Calculation of compatibility coefficient between evidences

Based on the basic idea of evidence support and evidence distance, this study believes that although there is a conflict between conflicting evidence, such a conflict is not absolute, and there must be consistent information that can be used effectively. Based on these ideas, this paper introduces the concept of inter-evidence compatibility coefficient. The definition is as follows.

Identify frame ${\varvec{\theta}}=\{{{\varvec{A}}}_{{\mathbf{1}}},{\text{A}}_{{\mathbf{2}}},\dots ,{{\varvec{A}}}_{{\varvec{n}}}\}$, take any $\forall {{\varvec{A}}}_{{\varvec{k}}}$, and BPAF of the two evidences are respectively ${{\varvec{m}}}_{{\varvec{i}}}({{\varvec{A}}}_{{\varvec{k}}})$ and ${{\varvec{m}}}_{{\varvec{j}}}({{\varvec{A}}}_{{\varvec{k}}})$, ${{\varvec{m}}}_{{\varvec{j}}}({{\varvec{A}}}_{{\varvec{k}}})$, then the compatibility coefficient of the two evidences with respect to ${{\varvec{A}}}_{{\varvec{k}}}$ is:

1 $${{\varvec{R}}}_{{\varvec{i}},{\varvec{j}}}({{\varvec{A}}}_{{\varvec{k}}})=\frac{{{\varvec{m}}}_{{\varvec{i}}}({{\varvec{A}}}_{{\varvec{k}}})*{{\varvec{m}}}_{{\varvec{j}}}({{\varvec{A}}}_{{\varvec{k}}})}{\frac{{\mathbf{1}}}{{\mathbf{2}}}({{\varvec{m}}}_{{\varvec{i}}}({{\varvec{A}}}_{{\varvec{k}}}{)}^{{\mathbf{2}}}+{{\varvec{m}}}_{{\varvec{j}}}({{\varvec{A}}}_{{\varvec{k}}}{)}^{{\mathbf{2}}})}$$

According to the above equation, when the BPA of any focal elements in ${{\varvec{m}}}_{{\varvec{i}}}({{\varvec{A}}}_{{\varvec{k}}})$ and ${{\varvec{m}}}_{{\varvec{j}}}({{\varvec{A}}}_{{\varvec{k}}})$ are zero, the value of ${{\varvec{R}}}_{{\varvec{i}},{\varvec{j}}}({{\varvec{A}}}_{{\varvec{k}}})$ is zero, indicating that in the evidence set, when one piece of evidence supports the focal element ${{\varvec{A}}}_{{\varvec{k}}}$, that is, when there is a high conflict between the two pieces of evidence, the compatibility coefficient is zero. When the BPA of ${{\varvec{m}}}_{{\varvec{i}}}({{\varvec{A}}}_{{\varvec{k}}})$ and ${{\varvec{m}}}_{{\varvec{j}}}({{\varvec{A}}}_{{\varvec{k}}})$ is equal, the ${{\varvec{R}}}_{{\varvec{i}},{\varvec{j}}}({{\varvec{A}}}_{{\varvec{k}}})$ value is 1, indicating that the compatibility coefficient is 1 in the evidence set when the support degree of the focal element ${{\varvec{A}}}_{{\varvec{k}}}$ of the two evidences is consistent, that is, when there is no conflict between the two evidences. Therefore, the compatibility coefficient can represent the degree of mutual support between two pieces of evidence.

The degree of consistency between the evidence in the evidence set can usually be expressed by the expected value, which can be expressed by the average evidence. Therefore, the higher the mutual support between the expected value and the evidence, the higher the credibility of the evidence and the corresponding weight should be.

Using the calculation idea of compatibility coefficient, when there are n pieces of evidence in the evidence set, the mutual support degree between each piece of evidence and the average evidence can be expressed as:

2 $${w}_{ik}\left({A}_{k}\right)={\overline{R}}_{i,k}\left({A}_{k}\right)=\frac{2{m}_{i}\left({A}_{k}\right)\overline{m}\left({A}_{k}\right)}{{m}_{i}({A}_{k}{)}^{2}+\overline{m}({A}_{k})},\,\,k=1,2,\dots ,n$$

where $\overline{{\varvec{m}}}({{\varvec{A}}}_{{\varvec{k}}})=\frac{{\mathbf{1}}}{{\varvec{n}}}{\sum }_{{\varvec{i}}={\mathbf{1}}}^{{\varvec{n}}}{{\varvec{m}}}_{{\varvec{i}}}({{\varvec{A}}}_{{\varvec{k}}})$ represents the average evidence, i.e. the overall expectation of the evidence.

It can be seen that the weight matrix of each evidence regarding each proposition is:

3 $$w=\left[\begin{array}{cccc}{\overline{R}}_{\mathrm{1,1}}({A}_{1})& {\overline{R}}_{\mathrm{1,2}}({A}_{2})& \cdots & {\overline{R}}_{1,n}({A}_{n})\\ {\overline{R}}_{\mathrm{2,1}}({A}_{1})& {\overline{R}}_{\mathrm{2,1}}({A}_{2})& \cdots & {\overline{R}}_{2,n}({A}_{n})\\ \vdots & \vdots & & \vdots \\ {\overline{R}}_{n,1}({A}_{1})& {\overline{R}}_{n,2}({A}_{2})& \cdots & {\overline{R}}_{n,n}({A}_{n})\end{array}\right]$$

Then, each focal element of BPA in each piece of evidence is redistributed using the above weights, namely:

4 $${{{\varvec{m}}}_{{\varvec{i}}}}^{{{\prime}}}({{\varvec{A}}}_{{\varvec{k}}})={\overline{{\varvec{R}}}}_{{\varvec{i}},{\varvec{k}}}({{\varvec{A}}}_{{\varvec{k}}}){{\varvec{m}}}_{{\varvec{i}}}({{\varvec{A}}}_{{\varvec{k}}})$$

BPA distribution for focal elements of evidence is as follows:

5 $${{{\varvec{m}}}_{{\varvec{i}}}}^{{{\prime}}}({\varvec{\theta}})={\mathbf{1}}-\sum\limits_{{\varvec{k}}={\mathbf{1}}}^{{\varvec{n}}}\left({\mathbf{1}}-{\overline{{\varvec{R}}}}_{{\varvec{i}},{\varvec{k}}}({{\varvec{A}}}_{{\varvec{k}}})\right){{\varvec{m}}}_{{\varvec{i}}}({{\varvec{A}}}_{{\varvec{k}}}),\,\,\,{\varvec{i}}={\mathbf{1}},{\mathbf{2}},\dots ,{\varvec{n}}$$

### Reliability calculation among evidences

Set the evidence set, ${{\varvec{m}}}_{{\mathbf{1}}},{{\varvec{m}}}_{{\mathbf{2}}},\dots ,{{\varvec{m}}}_{{\varvec{n}}},$ and set the focal element conflict size between winning game I and J as ${{\varvec{k}}}_{{\varvec{i}}{\varvec{j}}}$, then:

6 $${{\varvec{k}}}_{{\varvec{i}}{\varvec{j}}}=\sum\limits_{{\varvec{A}}{\varvec{i}}\cap {\varvec{A}}{\varvec{j}}=\boldsymbol{\varnothing }}{{\varvec{m}}}_{{\varvec{i}}}\left({{\varvec{A}}}_{{\varvec{i}}}\right){{\varvec{m}}}_{{\varvec{j}}}\left({{\varvec{A}}}_{{\varvec{j}}}\right)$$

Kij is the size of conflict between every two evidences in the evidence set, which reflects the sum of the magnitude of conflict between a certain focal element in evidence i and other focal elements in evidence j, and can effectively reflect the degree of conflict between evidences.

c is defined as the average conflict degree of the evidence set, then:

7 $${\varvec{c}}=\frac{{\mathbf{1}}}{{\varvec{n}}({\varvec{n}}-{\mathbf{1}})/{\mathbf{2}}}\sum\limits_{{\varvec{i}}<{\varvec{j}}}{{\varvec{k}}}_{{\varvec{i}}{\varvec{j}}},{\varvec{i}},{\varvec{j}}\le {\varvec{n}}$$

where ${\varvec{n}}$ is the number of evidence sources.

${\varvec{\varepsilon}}$ is defined as the credibility of evidence, then:

8 $${\varvec{\varepsilon}}=\frac{{\mathbf{1}}}{{{\varvec{e}}}^{{\varvec{c}}}}$$

where c is defined in Eq. (7).

Analysis of Eqs. (6) and (7) shows that ${\varvec{\varepsilon}}$ is the decreasing function of c. When the conflict among evidences increases, when C increases, the credibility of evidences will decrease. Therefore, ${\varvec{\varepsilon}}$ can represent the credibility of evidences.

### Improvement of fusion rules

This paper introduces inter-evidence credibility and improves Dempster's rule of combination as follows:

9 $${\varvec{p}}({\varvec{A}})=\sum\limits_{\begin{array}{c}{{\varvec{A}}}_{{\varvec{i}}}\in {{\varvec{M}}}_{{\varvec{i}}}\\ {\cap }_{{\varvec{i}}={\mathbf{1}}}^{{\varvec{n}}}{{\varvec{A}}}_{{\varvec{i}}}=A\end{array}}{{\varvec{m}}}_{{\mathbf{1}}}({{\varvec{A}}}_{{\mathbf{1}}}){{\varvec{m}}}_{{\mathbf{2}}}({{\varvec{A}}}_{{\mathbf{2}}})\cdots {{\varvec{m}}}_{{\varvec{n}}}({{\varvec{A}}}_{{\varvec{n}}}),{\varvec{q}}({\varvec{A}})$$

10 $$\left\{\begin{array}{l}m(A)=P(A)+k*\varepsilon *(q(A)+G(A)),A\ne \theta \\ m(\theta )=P(\theta )+k*\varepsilon *q(\theta )/(k+\varepsilon )\end{array}\right.$$

where ${\varvec{P}}({\varvec{A}})$, ${\varvec{Q}}({\varvec{A}})$ are Eq. (9), K is the discrepancy factor, is Eq. (8), ${\varvec{G}}({\varvec{A}})$ is the amount of evidence that can be trusted to A in an unknown focal element.

11 $${\varvec{G}}({\varvec{A}})=\sum\limits_{\begin{array}{c}{{\varvec{A}}}_{{\varvec{i}}}\in {{\varvec{M}}}_{{\varvec{i}}}\\ {\varvec{i}}\ne {\varvec{j}}\end{array}}{{\varvec{m}}}_{{\varvec{i}}}({\varvec{A}}){{\varvec{m}}}_{{\varvec{j}}}({\varvec{\theta}}){{\varvec{m}}}_{{\varvec{j}}}({\varvec{\theta}})\cdots {{\varvec{m}}}_{{\varvec{n}}}({\varvec{\theta}})/{\varvec{n}}$$

According to Eq. (10), when k is small, ${\varvec{P}}({\varvec{A}})$ plays major role in the equation, and the resultant result is similar to that of DS evidence theory. When k = 0, the new combination equation is the same as Dempster's combination rule. When K → 1, the combination result will be mainly determined by ${\varvec{\varepsilon}}{*}({\varvec{q}}({\varvec{A}})+{\varvec{G}}({\varvec{A}}))$. Therefore, when the evidence is highly conflicting, the fusion result of the improved Dempster combination rule will be mainly determined by ${\varvec{\varepsilon}}{*}({\varvec{q}}({\varvec{A}})+{\varvec{G}}({\varvec{A}}))$.

Against sun quan combination rules, the k increases or ${\varvec{\varepsilon}}$ decreases, and ${\varvec{k}}({\mathbf{1}}-{\varvec{\varepsilon}})$ will increase, which in turn leads to increasing synthetic results of unknown problems. In this paper, the BPAF of unknown focal element is improved. According to ${\varvec{m}}({\varvec{\theta}})$ in Eq. (10), when the inconsistency factor K increases or the evidence credibility ${\varvec{\varepsilon}}$ decreases, ${\varvec{k}}\mathbf{*}{\varvec{\varepsilon}}/({\varvec{k}}+{\varvec{\varepsilon}})$ can more reasonably balance the conflict between K and ${\varvec{\varepsilon}}$.

### Steps of conflict evidence processing and fusion

This study improves the DS evidence theory from two aspects. On the one hand, it introduces the compatibility coefficient to assign different weights to the focal elements that cause conflicts in the consistency information among evidences, so as to minimize the influence of evidence with low reliability on the fusion results. For evidence, on the other hand, the conflict between information, introducing the credibility of evidence, the evidence for integration, support for highly give fully affirmation of evidence, the evidence than major, of low trust evidence for the separation, to make it small in proportion of evidence, in highly conflict evidence, greatly reduce the uncertainty of the fusion results, reduce the correlation between low interference in the process of integration of evidence, makes the result more clear and reasonable.

**Figure 1:**
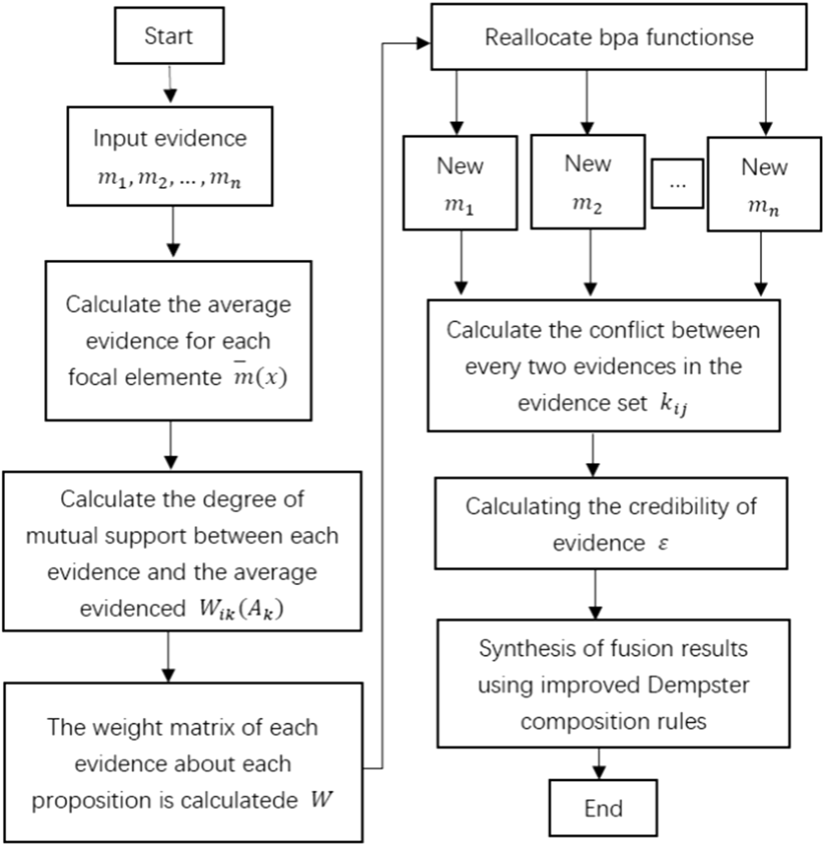
Conflict evidence processing flow chart.

According to above analysis, the processing of conflict evidence in this study should be started with revised data first. Then it will use compatible coefficient to modify source of conflict evidence. After the new source of evidence is get, using introduction credibility of evidence and evidence focal element average support synthesis rules in the new source of conflict evidence combination of evidence, concrete evidence conflict process is shown in Fig. 1.

It can be seen from the above processing process that the steps of processing conflict evidence are summarized as follows:

Step 1: Calculate the weight coefficient corresponding to each focal element in each piece of evidence by Formula (2);

Step 2: According to the weight coefficient of each focal element in each evidence, BPAF is redistributed according to Eqs. (4) and (5) to obtain a new BPA;

Step 3: Calculate the conflicts between every two pieces of evidence in the evidence set ${{\varvec{k}}}_{{\varvec{i}}{\varvec{j}}}$ and ${\varvec{\varepsilon}}$;

Step 4: Obtain the fusion result through Eq. (9).

## Experimental comparison and example verification

### Experimental comparison of the synthesis of conflicting evidence

In this paper, Dempster's rule, Murphy's method, Yager method, Sun Quan method and this research method were respectively used to fuse conflicting evidence of the four problems existing in D–S evidence theory, and the fusion results were compared and analyzed to verify the effectiveness of this algorithm. Taking the following evidence as an example, the evidence includes general conflict evidence, focal element rejection evidence, focal element fine-tuning evidence and complete conflict evidence.

Set the identification framework ${\varvec{\theta}}=\left\{{\varvec{A}},{\varvec{B}},{\varvec{C}}\right\}$, and the evidence is as follows:

$$\begin{aligned}{{\varvec{m}}}_{{\mathbf{1}}}{:}\,\,{{\varvec{m}}}_{{\mathbf{1}}}({\varvec{A}})&={\mathbf{0.99}},\,\,{{\varvec{m}}}_{{\mathbf{1}}}({\varvec{B}})={\mathbf{0.01}},\,\,{{\varvec{m}}}_{{\mathbf{1}}}({\varvec{C}})={\mathbf{0}},\,\,{{\varvec{m}}}_{{\mathbf{1}}}({\varvec{\theta}})={\mathbf{0}}\\{{\varvec{m}}}_{{\mathbf{2}}}{:}\,\,{{\varvec{m}}}_{{\mathbf{2}}}({\varvec{A}})&={\mathbf{0}},\,\,{{\varvec{m}}}_{{\mathbf{2}}}({\varvec{B}})={\mathbf{0.01}},\,\,{{\varvec{m}}}_{{\mathbf{1}}}({\varvec{C}})={\mathbf{0.99}},\,\,{{\varvec{m}}}_{{\mathbf{2}}}({\varvec{\theta}})={\mathbf{0}}\\{{\varvec{m}}}_{{\mathbf{3}}}{:}\,\,{{\varvec{m}}}_{{\mathbf{3}}}({\varvec{A}})&={\mathbf{0.98}},\,\,{{\varvec{m}}}_{{\mathbf{3}}}({\varvec{B}})={\mathbf{0.01}},\,\,{{\varvec{m}}}_{{\mathbf{3}}}({\varvec{C}})={\mathbf{0.01}},\,\,{{\varvec{m}}}_{{\mathbf{3}}}({\varvec{\theta}})={\mathbf{0}}\\{{\varvec{m}}}_{{\mathbf{4}}}{:}\,\,{{\varvec{m}}}_{{\mathbf{4}}}({\varvec{A}})&={\mathbf{0}},\,\,{{\varvec{m}}}_{{\mathbf{4}}}({\varvec{B}})={\mathbf{1}},\,\,{{\varvec{m}}}_{{\mathbf{4}}}({\varvec{C}})={\mathbf{0}},\,\,{{\varvec{m}}}_{{\mathbf{4}}}({\varvec{\theta}})={\mathbf{0}}\\{{\varvec{m}}}_{{\mathbf{5}}}{:}\,\,{{\varvec{m}}}_{{\mathbf{5}}}({\varvec{A}})&={\mathbf{1}},\,\,{{\varvec{m}}}_{{\mathbf{5}}}({\varvec{B}})={\mathbf{0}},\,\,{{\varvec{m}}}_{{\mathbf{5}}}({\varvec{C}})={\mathbf{0}},\,\,{{\varvec{m}}}_{{\mathbf{5}}}({\varvec{\theta}})={\mathbf{0}}\end{aligned}$$

In the evidence set, ${\varvec{m}}({\varvec{A}}),{\varvec{m}}({\varvec{B}}),{\varvec{m}}({\varvec{C}})$ respectively represent BPAF as the BPA allocated to identify each focal element in the framework, and ${\varvec{m}}({\varvec{\theta}})$ represents the probability of assigning evidence to unknown focal elements.

#### For general conflict problems

The evidence collection of ${{\varvec{m}}}_{{\mathbf{1}}},{{\varvec{m}}}_{{\mathbf{2}}}$ shows that when general conflict problem, that is when focal elements A and C of BPA in evidence m1 were 0.99 and 0, focal elements ${\varvec{A}}$ and ${\varvec{C}}$ of BPA in evidence ${{\varvec{m}}}_{{\mathbf{2}}}$ were 0 and 0.99 respectively. Obviously, the two evidences are conflicting, and coefficient of evidence conflict approaches to be 1, and the fusion results of focal element A and C should be equal, but not 0. From the Table 1, the fusion results by Dempster rule and Yager method assign focal element A and C to 0, so these two methods cannot effectively fusion data conflict, But Murphy, Sun Quan method and the method in this paper can solve the problem of general conflict to some extent, but Murphy method only make weighted average to conflict simply, and the convergence speed is slow. The value of ${\varvec{m}}({\varvec{A}})$ and ${\varvec{m}}({\varvec{C}})$ in the fusion results of the Sun Quan method are not zero, but the weight which the conflict evidence divided into the unknown field is higher in this method, the ${\varvec{m}}({\varvec{\theta}})$ value is higher than the values of ${\varvec{m}}({\varvec{A}})$ and ${\varvec{m}}({\varvec{C}})$, it could not judge the fusion results, so the fusion results failed to meet expectations. In this paper, ${\varvec{m}}({\varvec{A}})$ and ${\varvec{m}}({\varvec{C}})$ values are not 0, and the weight of evidence divided into unknown focal elements is small, which conforms to the expected results. Therefore, the method of this study can solve the general conflict problem to a certain extent.

**Table 1:** The processing results of different algorithms for general conflict problems.

${m}_{1},{m}_{2}$	$m(A)$	$m(B)$	$m(C)$	$m(\theta )$	$k$
Dempster^16^	0	1	0	0	0.9999
Murphy^17^	0.4999	0.0002	0.3985	0	0.5099
Yager^18^	0	0.0001	0.0005	0.9999	0.9999
Sun^19^	0.1821	0.0038	0.4701	0.6320	0.9999
This paper	0.4581	0.0107	0.3424	0.0733	0.6432

#### The question of one veto

From the evidence sets ${{\varvec{m}}}_{{\mathbf{1}}},{{\varvec{m}}}_{{\mathbf{2}}},{{\varvec{m}}}_{{\mathbf{3}}}$, the BPA of the focal element A in ${{\varvec{m}}}_{{\mathbf{1}}}$ and ${{\varvec{m}}}_{{\mathbf{3}}}$ is 0.99 and 0.98 respectively, and the BPA of the focal element A in ${{\varvec{m}}}_{{\mathbf{2}}}$ is 0. Obviously, although the evidence sets ${{\varvec{m}}}_{{\mathbf{1}}}$ and ${{\varvec{m}}}_{{\mathbf{3}}}$ both support the focal element A, the evidence sets M2 opposes the focal element A. As can be seen from Table 2, the fusion results of Dempster's rule and Yager method both have 0 assignment of focal element A, and the fusion results are not as expected. So these two methods cannot effectively fuse conflicting evidence. The values of ${\varvec{m}}({\varvec{A}})$ in Murphy, Sun Quan method and the method in this paper are not zero, so can solve the Veto problems to some extent, but Murphy method only make weighted average to conflict simply, and the convergence speed is slow, the value of ${\varvec{m}}({\varvec{\theta}})$ in fusion result of Sun Quan is still the highest values, unable to judge the fusion results, so the fusion results failed to meet expectations. The ${\varvec{m}}({\varvec{A}})$ value obtained in the method in this paper is the highest, and the fusion target points to the focal element A, which meets the expected result. Therefore, the method of this study can solve the problem of one vote veto to some extent.

**Table 2:** Different algorithms deal with the problem of one vote rejection.

${m}_{1},{m}_{2}$	$m(A)$	$m(B)$	$m(C)$	$m(\theta )$	$k$
Dempster	0	1	0	0	1
Murphy	0.8848	0	0.1152	0	0.6795
Yager	0	0	0	1	1
Sun	0.3349	0.0051	0.1700	0.4900	1
This paper	0.7722	0.0071	0.1575	0.0636	0.6120

#### Robustness

The evidence collection of ${{\varvec{m}}}_{{\mathbf{2}}},{{\varvec{m}}}_{{\mathbf{3}}}$ shows that the BPA of the focal element A in m3 is 0.98, which is reduced 0.01 than BPA of the focal element A in m1 focal element A. But focal elements BPA from Dempster rule that is compared between the Tables 1 and Table 3, shows that the fusion results change from pointing to focal element B to the focal element C. This method takes great changes in the recognition of the target object, so it can not the conflict evidence for effective synthesis. The results of Murphy method, Yager method, Sun Quan method and the method in this paper are not much different from the results of dealing with general conflict problems. Therefore, the method in this paper can solve the robustness problem to some extent.

**Table 3:** The processing results of different algorithms for robustness problems.

${m}_{1},{m}_{2}$	$m(A)$	$m(B)$	$m(C)$	$m(\theta )$	$k$
Dempster	0	0.01	0.99	0	0.99
Murphy	0.4898	0.0002	0.6100	0	0.5098
Yager	0	0.0001	0.0099	0.99	0.99
Sun	0.1803	0.0038	0.1938	0.6221	0.99
This paper	0.4493	0.0109	0.2677	0.0725	0.6406

#### Rule failure problem

From the evidence sets ${{\varvec{m}}}_{{\mathbf{4}}},{{\varvec{m}}}_{{\mathbf{5}}}$, it can be known that the values of inconsistent factor k in m4 and m5 are all 1, that is, evidence ${{\varvec{m}}}_{{\mathbf{4}}},{{\varvec{m}}}_{{\mathbf{5}}}$ completely conflict, but the BPA of focal element B in evidence m4 and the BPA of focal element A in evidence ${{\varvec{m}}}_{{\mathbf{5}}}$ are 1, so the fusion results should point to focal element A and focal element B on average. It can be seen from Table 4 that Dempster's rule cannot be used in this case. The Yager method completely allocates the conflict result to the unknown focal element, and the obtained result is inconsistent with the actual situation. Murphy method, Sun Quan method and the method in this paper can solve the rule failure problem to some extent, and the obtained result points to the BPA of focal element A and focal element B. However, Murphy's method only made a simple weighted average of the conflicting evidence. The weight of the Sun Quan method divided the conflicting evidence into unknown fields was higher than other focal elements, which failed to effectively identify the target object. The value of ${\varvec{m}}({\varvec{\theta}})$ obtained by the method in this paper was small, and the ${\varvec{m}}({\varvec{A}})$ and ${\varvec{m}}({\varvec{B}})$ values met the expected results. Therefore, the method in this paper can solve the problem of rule failure to some extent.

**Table 4:** The processing results of different algorithms to rule failure problem.

$m_{1} ,m_{2}$	$m(A)$	$m(B)$	$m(C)$	$m(\theta )$	$k$
Dempster	–	–	–	–	–
Murphy	0.5	0.5	0	0	0.5
Yager	0	0	0	1	1
Sun	0.1839	0.3653	0	0.6322	1
This paper	0.4976	0.8324	0	0.006	0.6401

## Conclusion

In this paper, based on the analysis of the classical evidence combination rule improvement algorithm, an improved DS evidence theory synthesis algorithm is studied and designed for the problems of general conflict, one-vote veto, poor robustness and synthesis rule failure of DS evidence theory algorithm. The algorithm introduces the concepts of compatibility coefficient and credibility, and uses the compatibility coefficient to correct the original evidence on the one hand, and the credibility to improve the Dempster combination rule on the other hand, and finally verifies the effectiveness of the algorithm in this paper through experimental comparison and example verification. The experimental results show that: compared with other algorithms, the algorithm in this paper can solve the problems of classical D–S evidence theory to a certain extent, and the inferred fusion results are more reasonable and converge faster.
